# Quantitative hormone receptor (HR) expression and gene expression analysis in HR+ inflammatory breast cancer (IBC) vs non-IBC

**DOI:** 10.1186/s12885-020-06940-z

**Published:** 2020-05-18

**Authors:** Toshiaki Iwase, Kenichi Harano, Hiroko Masuda, Kumiko Kida, Kenneth R. Hess, Ying Wang, Luc Dirix, Steven J. Van Laere, Anthony Lucci, Savitri Krishnamurthy, Wendy A. Woodward, Rachel M. Layman, François Bertucci, Naoto T. Ueno

**Affiliations:** 1 Morgan Welch Inflammatory Breast Cancer Research Program and Clinic, The University of Texas MD Anderson Cancer Center, 1515 Holcombe Blvd, Houston, TX 77030 USA; 2 Section of Translational Breast Cancer Research, Department of Breast Medical Oncology, The University of Texas MD Anderson Cancer Center, 1515 Holcombe Blvd, Houston, TX 77030 USA; 3 Department of Biostatistics, The University of Texas MD Anderson Cancer Center, 1515 Holcombe Blvd, Houston, TX 77030 USA; 4 Department of Bioinformatics and Computational Biology, The University of Texas MD Anderson Cancer Center, 1515 Holcombe Blvd, Houston, TX 77030 USA; 5 Department of Oncology, University of Antwerp, Prinsstraat 13, 2000 Antwerpen, Belgium; 6 Department of Breast Surgical Oncology, The University of Texas MD Anderson Cancer Center, 1515 Holcombe Blvd, Houston, TX 77030 USA; 7 Department of Anatomical Pathology, The University of Texas MD Anderson Cancer Center, 1515 Holcombe Blvd, Houston, TX 77030 USA; 8 Department of Radiation Oncology, The University of Texas MD Anderson Cancer Center, 1515 Holcombe Blvd, Houston, TX 77030 USA; 9 Department of Breast Medical Oncology, The University of Texas MD Anderson Cancer Center, 1515 Holcombe Blvd, Houston, TX 77030 USA; 10 Laboratory of Predictive Oncology, Centre de Recherche en Cancérologie de Marseille (CRCM), Inserm, U1068, CNRS UMR7258, Institut Paoli-Calmettes, Aix-Marseille Université, F-13009 Marseille, France

**Keywords:** Inflammatory breast neoplasms, Estrogen receptors, Immunohistochemistry, Gene expression

## Abstract

**Background:**

The purpose of this study was to determine the prognostic role of hormone receptor (HR) on inflammatory breast cancer (IBC) to elucidate its aggressive biological behavior.

**Methods:**

We evaluated the expression of estrogen receptor (ER) and progesterone receptor (PR) by immunohistochemical staining and determined the predictive and prognostic role of HR expression on 189 patients with HR+/HER2– IBC and 677 patients with HR+/HER2– stage III non-IBC. Furthermore, we performed gene expression (GE) analyses on 137 patients with HR+/HER2– IBC and 252 patients with HR+/HER2– non-IBC to detect genes that are specifically overexpressed in IBC.

**Results:**

The expression of ER% was significantly associated with longer distant disease-free survival and overall survival. However, there was no significant relationship between ER% and neoadjuvant chemotherapy outcome. In the GE study, 84 genes were identified as significantly distinguishing HR+ IBC from non-IBC. Among the top 15 canonical pathways expressed in IBC, the ERK/MAPK, PDGF, insulin receptor, and IL-7 signaling pathways were associated with the ER signaling pathway. Upregulation of the *MYC* gene was observed in three of these four pathways. Furthermore, HR+/HER2– IBC had significantly higher *MYC* amplification, and the genetic alteration was associated with poor survival outcome.

**Conclusions:**

Higher ER expression was significantly associated with improved survival in both HR+/HER2– IBC and HR+/HER2– stage III non-IBC patients. HR+/HER2– IBC had several activated pathways with *MYC* upregulation, and the genetic alteration was associated with poor survival outcome. The results indicate that *MYC* may be a key gene for understanding the biology of HR+/HER2– IBC.

## Background

Inflammatory breast cancer (IBC) is a rare type of breast malignancy characterized by diffuse erythema and edema called *peau d’orange* without palpable mass. The incidence is approximately 2.0 to 2.5% in a U.S. national survey [1]. This phenotype is also known to have a very aggressive tumor behavior, with a 2.9- to 4.2-year median survival period, which is a significantly poorer survival period than that in locally advanced non-IBC [1, 2].

Estrogen receptor (ER) and progesterone receptor (PR) expression by immunohistochemical (IHC) analysis is commonly used as a predictive marker for endocrine treatment as well as a prognostic indicator in non-IBC [3, 4]. Commonly, ER and PR expression by IHC analysis is positively associated with response to endocrine treatment and with better prognosis in early-stage ER-positive (ER+) non-IBC [3, 5]. However, the role of these hormone receptors (HRs) in IBC has been inconsistent, according to a retrospective analysis based on a large data registry [6, 7].

We previously investigated the prognostic value of HRs in patients with IBC who underwent neoadjuvant chemotherapy (NAC) and found that HR positivity had no prognostic value for survival after NAC among HR-positive (HR+)/human epidermal growth factor receptor 2–positive (HER2+), HR+/HER2-negative (HER2−), and HR-negative (HR−)/HER2+ subtypes [8]. This result was in contrast to those of previous studies showing that the ER+/HER2− subtype demonstrated significantly worse survival outcome compared with ER+/HER2+ or ER-negative (ER−)/HER2+ subtypes [7], or that ER positivity had a significant association with better survival outcome in patients with IBC, regardless of the type of treatment [6]. Although these inconsistencies may be explained by the nature of retrospective analysis, more detailed analysis is needed to understand the mechanism responsible for the differences between HR+ IBC and non-IBC. To elucidate this mechanism, we applied a two-step approach—an IHC analysis and gene expression (GE) analysis focused on the estrogen signaling pathway in IBC.

Our main hypothesis was that HR expression has a prognostic role in HR+/HER2– IBC and that HR+/HER2– IBC has specific GE in the ER signaling pathway that characterizes aggressive biological behavior.

## Methods

### Patient selection

Our study population consisted of two groups: (1) the IHC study group, which consisted of 866 patients (189 IBC and 677 non-IBC) and (2) the GE study group, which included 389 patients (137 IBC and 252 non-IBC).

#### IHC study

For the IHC study group, we retrospectively reviewed clinical and pathological information from the breast cancer electronic medical record management system at The University of Texas MD Anderson Cancer Center between January 1, 1989, and April 30, 2015 (*n* = 1731). A multidisciplinary team, consisting of a medical oncologist, surgical oncologist, radiologist, and nurse, determined the clinical diagnosis of IBC according to the IBC-specific clinical manifestation. This clinical manifestation includes history of rapid onset of breast erythema, edema and/or peau d’orange, and/or warm breast, with or without an underlying palpable mass. A history of flattening, crusting, or retraction of the nipple were also considered. We excluded cases with inflammatory skin change secondary to non-IBC.

For patient selection, we first excluded patients who did not have adequate pathological information with which to determine the percentage expression of ER (ER%) and the percentage expression of PR (PR%) (*n* = 452). Next, we excluded patients who had undergone neoadjuvant endocrine therapy (*n* = 59), no definitive surgery (*n* = 43), or insufficient pathological data for pathological complete response (pCR) (*n* = 25) or survival (*n* = 11). We also excluded patients with T stage 0–2 (*n* = 275) because we did not consider these stages to be locally advanced. Finally, we obtained 866 eligible patients, including 189 with IBC and 677 with case-matched stage III non-IBC (Supplementary Fig. 1).

#### GE study

For the GE study group, we used mRNA expression data from the World IBC Consortium gene database [9]. The World IBC Consortium is a multicenter collaborative project that explores the biology of IBC based on gene expression by applying whole-transcriptome Affymetrix DNA microarrays. This data set includes the comprehensive gene set used in our study of 137 IBC patients and 252 non-IBC patients.

### Data collection

#### Pathological evaluation for IHC study

We obtained the continuous value of percentage HR expression both in ER and PR. We defined ER as positive if ER expression by IHC was 1% or more. HER2 positivity was determined according to the ASCO/CAP guidelines at the time of pathological evaluation. We defined pCR as no invasive components in residual tumor in the primary site or axillary lymph nodes in the surgical specimen [10].

#### GE evaluation and pathway analysis for GE study

We examined GE differences between patients with HR+/HER2– IBC and HR+/HER2– non-IBC by feature-by-feature linear mixture models and then fitting a beta-uniform mixture model to control for multiple testing [11, 12]. The number of significant genes was counted for false discovery rates at 1%. We used the Affymetrix U133 annotation package hgu133a.db (Affymetrix, Santa Clara, CA, USA) to export gene symbols for 22,283 probes. We determined upregulation and downregulation by median value of gene expression. After identifying significantly upregulated/downregulated genes in IBC, we looked for enriched canonical pathways that included these genes by using ingenuity pathway analysis (QIAGEN, Germantown, MD, USA). Next, we investigated the relationship between these canonical pathways and the ER signaling pathway.

### Statistical analysis

We compared the clinicopathological characteristics between patients with HR+/HER2– IBC and corresponding non-IBC with use of a chi-square test for categorical data and Student *t* test for interval-scaled data. We also used a logistic regression model to determine the association between ER%, PR%, and pCR.

#### Survival analysis and setting the cutoff points

We performed a survival analysis with two outcomes for the IHC study (distant disease-free survival [DDFS] and overall survival [OS]) and three outcomes for the GE study (recurrence-free survival [RFS], DDFS, and OS). We defined RFS as the time from the date of definitive surgery to the date of locoregional recurrence or distant metastasis, DDFS as the time from the date of definitive surgery to the date of distant metastasis, and OS as the time from the date of definitive surgery to the date of death due to any causes or the date of last follow-up. Survival rates were calculated by using the Kaplan-Meier method, and curves were compared with the log-rank test. In the Cox proportional hazard model, we adjusted for age, menopausal status, histology, cN stage, cT stage, lymphatic invasion, vascular invasion, grade, and mastectomy status. We calculated the hazard ratio for HR expression as 50% increase, which can be thought of as comparing outcomes in two patients, one with ER/PR level X and another with ER/PR level X + 50%. We applied recursive partitioning analysis (RPA) to determine the optimal cutoff points for ER% and PR% that maximized the difference in DDFS. RPA created a regression tree that was divided by certain cutoff points that maximized the difference in outcome and then determined the optimal cutoff points [13].

In addition, we performed an external validation analysis by using an external cohort from the Institut Paoli-Calmettes (Marseille, France). The cohort included 57 patients with HR+/HER2– IBC and 78 patients with stage III HR+/HER2– non-IBC who underwent NAC between February 1, 1993, and February 28, 2015. All statistical analyses were performed two-sided, and *P* < 0.05 was defined as statistically significant. This study was approved by the Institutional Review Board at MD Anderson Cancer Center (PA17–0491).

## Results

### IHC analysis

#### Patient characteristics

Patients with IBC demonstrated significantly higher nuclear grades (*P* < 0.001) and more frequent ductal histology than did those in the non-IBC group (*P* = 0.003). In contrast, positivity for lymphatic and vascular invasion was not significantly different between the non-IBC and the IBC groups. Significantly more patients in the non-IBC group received adjuvant endocrine therapy than did patients in the IBC group (*P* = 0.007, Table 1). There were no significant differences regarding to the radiation therapy between two groups. The Mann-Whitney U test showed that the IBC group had significantly lower ER% and PR% compared with the non-IBC group (median ER%: 85% for IBC vs. 90% for non-IBC, *P* = 0.012; median PR%: 30% for IBC vs. 50% for non-IBC, *P* = 0.034) (Supplementary Fig. 2).

**Table 1:** Patient characteristics

Characteristic		non-IBC No.		IBC No.		
		(*n* = 677)	%	(*n* = 189)	%	*P*
Age, median (range), y		50 (22–83)		51 (23–75)		0.56
BMI, mean ± SD, m^2^		29.3 ± 6.8		31.7 ± 7.5		0.93
Menopausal status						0.002
	Pre	352	(52)	75	(40)	
	Post	304	(45)	108	(57)	
	Unknown	21	(3)	6	(3)	
cT stage						< 0.001
	3	395	(58)	0	(0)	
	4	282	(42)	189	(100)	
	Unknown	0	(0)	0	(0)	
cN stage						0.046
	0	57	(8)	29	(15)	
	1	415	(61)	109	(58)	
	2	69	(10)	18	(10)	
	3	135	(20)	33	(17)	
	Unknown	1	(1)	0	(0)	
Histology						0.003
	Ductal	507	(75)	160	(85)	
	Lobular	103	(15)	12	(6)	
	Mixed	46	(7)	9	(5)	
	Other	21	(3)	8	(4)	
Histological grade						< 0.001
	1	46	(7)	4	(2)	
	2	313	(46)	67	(35)	
	3	288	(43)	107	(57)	
	Unknown	30	(4)	11	(6)	
Lymphatic invasion						0.277
	Positive	389	(57)	116	(61)	
	Negative	271	(40)	67	(36)	
	Unknown	17	(3)	6	(3)	
Vascular invasion						0.512
	Positive	400	(59)	106	(56)	
	Negative	260	(38)	77	(41)	
	Unknown	17	(3)	6	(3)	
NAC regimen						0.308
	A	74	(11)	27	(14)	
	T	28	(4)	5	(2)	
	A + T	572	(84)	156	(83)	
	Other	3	(1)	1	(1)	
Adjuvant chemotherapy						0.001
	Yes	120	(13)	54	(28)	
	No	557	(87)	135	(72)	
Adjuvant endocrine therapy						0.007
	Yes	590	(87)	150	(79)	
	No	87	(13)	39	(21)	
Neoadjuvant radiation therapy						0.056
	Yes	6	(1)	5	(3)	
	No	671	(99)	184	(97)	
Adjuvant radiation therapy						0.100
	Yes	612	(90)	163	(86)	
	No	65	(10)	26	(14)	

Abbreviations; *IBC* inflammatory breast cancer, *BMI* body mass index, *SD* standard deviation, *NAC* neoadjuvant chemotherapy, *A* anthracycline, *T* taxane

#### Treatment response, survival analysis, and HR expression

Of 677 study patients with non-IBC, 33 (5%) achieved pCR after NAC; of 189 patients with IBC, 13 (7%) achieved pCR. Our logistic regression model showed that the ER% and PR% were not significantly associated with pCR in either non-IBC or IBC (data not shown). The median follow-up for non-IBC and IBC was 4.0 and 3.8 years, respectively. During follow-up, 90 IBC patients (48%) and 226 non-IBC patients (33%) had distant recurrences; also during this period, 80 IBC patients (42%) and 186 non-IBC patients (27%) died.

**Fig. 1:**
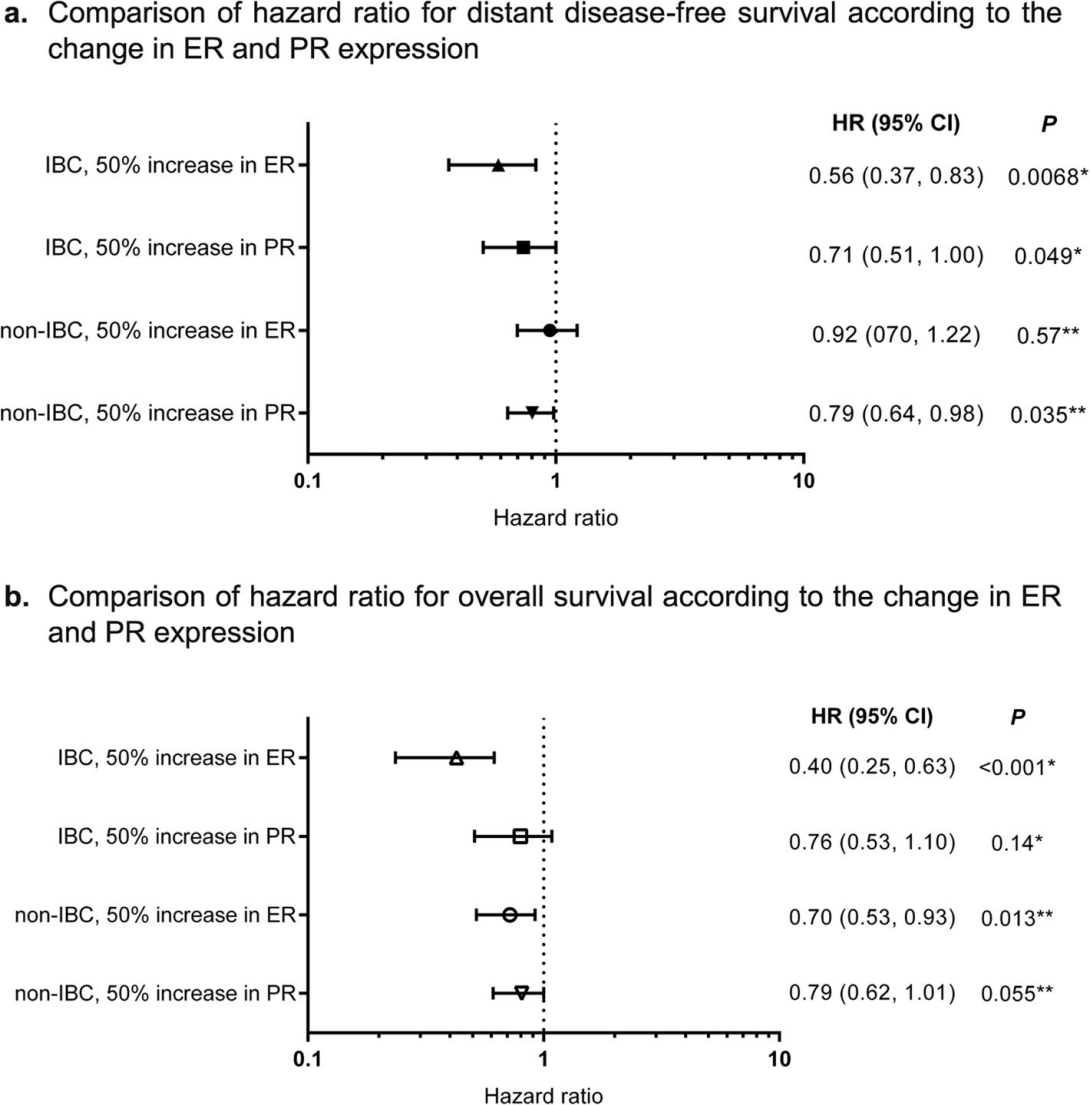
Effects of change in ER% and PR% on survival outcomes by multivariate analysis. **a** Comparison of hazard ratio for distant disease-free survival according to the change in ER and PR expression. **b** Comparison of hazard ratio for overall survival according to the change in ER and PR expression

In the multivariate analysis, expression of ER% was significantly associated with longer DDFS as well as OS for IBC (*P* = 0.0068 for DDFS and *P* < 0.001 for OS). However, the effect of the PR% was marginal or non-significant, respectively, for DDFS and OS (*P* = 0.049 for DDFS and *P* = 0.14 for OS) (Fig. 1). A similar association between ER expression and survival outcome was observed in non-IBC.

#### ER% and PR% cutoff points

**Fig. 2:**
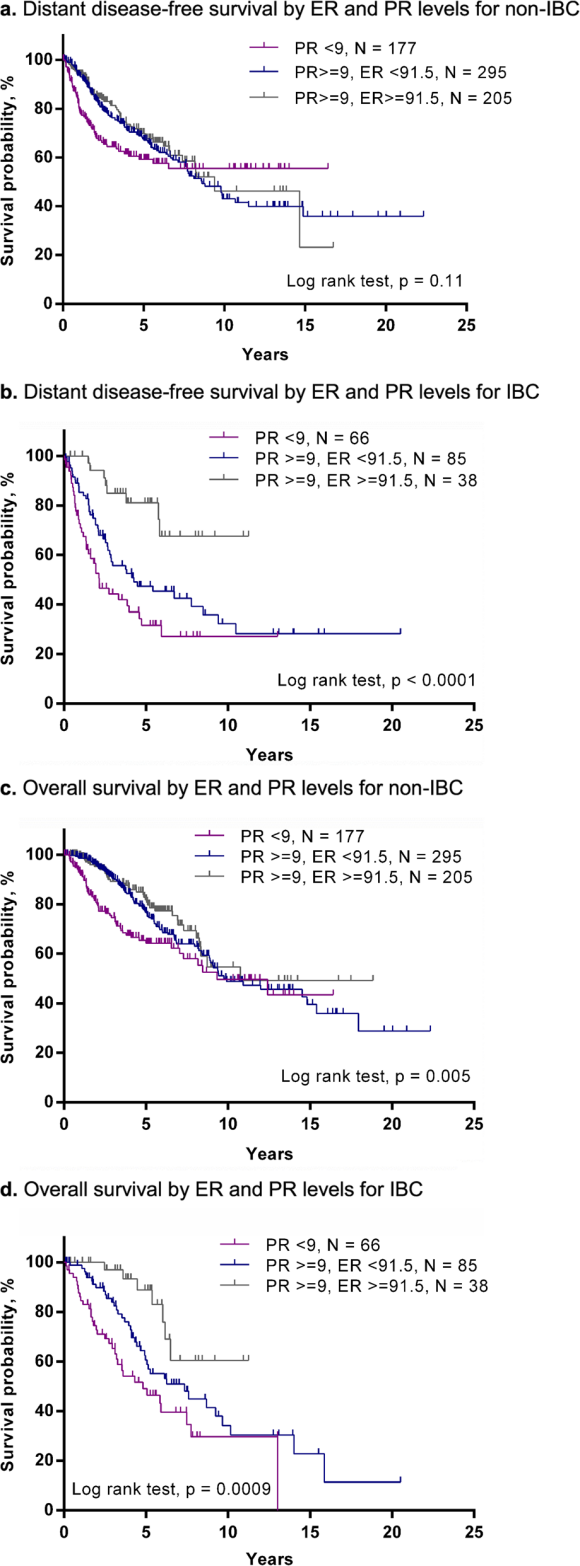
Survival outcomes according to newly defined cutoff points for ER and PR expression in ER+/HER2– IBC and corresponding non-IBC. **a** Distant disease-free survival by ER and PR levels for non-IBC. **b** Distant disease-free survival by ER and PR levels for IBC **c** Overall survival by ER and PR levels for non-IBC**. d** Overall survival by ER and PR levels for IBC

RPA showed that the optimal cutoff points for DDFS in ER% and PR% in IBC were 91.5 and 9%, respectively (Fig. 2b). The same cutoff points also distinguished OS for IBC (Fig. 2d). In non-IBC, the survival curves for the group with ER% ≥ 91.5% and PR% ≥ 9%, and for the group with ER% < 91.5% and PR% ≥9%, were overlapped for DDFS and OS (Fig. 2a and c).

We attempted an external validation of the newly established cutoff with use of the external cohort from Institut Paoli-Calmettes. The median follow-up periods for IBC and non-IBC patients were 7.0 and 9.0 years, respectively. During follow-up, 28 IBC patients (49%) and 23 non-IBC patients (30%) had distant recurrences; 20 IBC patients (35%) and 9 non-IBC patients (12%) died. DDFS and OS rates were lower in IBC than in non-IBC patients. Although a similar pattern of survival curves was observed in DDFS and OS for non-IBC group, the survival analysis with optimal grouping for ER% and PR% identified in the training set showed no significant differences in prognosis in the IBC group (Supplementary Fig. 3a-d).

### Gene expression analysis

Although the validation study on the outside cohort could not determine the universality of newly detected cutoff points, the IHC study suggested that ER% was associated with significantly better survival outcome in ER+/HER2– IBC than in corresponding non-IBC. This result indicated the difficulty in establishing universal cutoff points for HR+ IBC and the need to deeply investigate the role of the ER signaling pathway at the gene level. To this end, we further compared GE between HR+/HER2– IBC patients and the corresponding non-IBC patients to detect the specific genetic alteration in the ER signaling pathway.

#### Pathway analysis of significant genes associated with IBC status

The distribution of patient characteristics was not significantly different between the IBC and non-IBC groups (Supplementary Table 1). We identified 97 probe sets that significantly distinguished IBC from non-IBC at a false discovery rate of 1%. Among the 97 probe sets, 13 did not have a gene symbol in the Affymetrix annotation package, and 84 genes remained (Supplementary Table 2).

After the 84 genes associated with IBC were investigated with use of ingenuity pathway analysis, the top 15 canonical pathways in which these genes were included were revealed (Supplementary Fig. 4). However, the number of genes included in each pathway was relatively small (1 to 3). Among the top 15 pathways, the extracellular signal-regulated kinase (ERK)/mitogen-activated protein kinase (MAPK) signaling pathway, platelet-derived growth factor (PDGF) pathway, insulin receptor signaling pathway, and interleukin-7 (IL-7) signaling pathway overlapped with the ER signaling pathway. Among the upregulated/downregulated genes in those four pathways, *MYC* was the most frequently observed upregulated gene in three of the pathways (Supplementary Table 3).

#### Survival analysis based on MYC expression

The Wilcoxon test showed no significant differences in *MYC* expression levels between patients with HR+/HER2– IBC and those with non-IBC. In IBC patients, a Cox proportional hazard model indicated significant associations between *MYC* level and RFS (hazard ratio, 1.93; 95% confidence interval, 1.09 to 3.43, *P* = 0.003) and between *MYC* level and DDFS (hazard ratio, 2.00; 95% confidence interval, 1.10 to 3.64, *P* = 0.028), but not between *MYC* level and OS (hazard ratio, 1.45; 95% confidence interval, 0.65 to 3.24, *P* = 0.38) in HR+/HER2– IBC (Supplementary Table 4).

## Discussion

To the best of our knowledge, the present study was the first to find that the positivity level of ER expression had a significant prognostic role, even in HR+/HER2– IBC. Furthermore, the GE exploratory analyses indicated that *MYC* was the key gene in understanding the biological behavior of HR+/HER2– IBC.

In contrast to the predictive value, we identified the prognostic role of ER in HR+/HER2– IBC. Basically, the HR-positive breast cancer population shows a low percentage of pCR because of tumor dormancy, and as tumor stage becomes more advanced, pCR can be more difficult to obtain [7, 8]. Indeed, the present study showed that only 13 IBC patients (7%) and 33 non-IBC patients (5%) experienced pCR, which was very small compared with the population of patients with early breast cancer. Notably, patients with HR+/HER2– IBC had a wider range of heterogeneity in survival outcome according to ER expression level, and those with high ER expression had a better prognosis, which was similar to that of non-IBC patients. The results indicated that ER expression level also had an important prognostic role even in patients with HR+/HER2– IBC.

The present study also detected the optimal cutoff points for survival in HR+/HER2– IBC at 91.5% for ER and 9% for PR. Furthermore, these cutoff points were IBC-specific since they could not be applied to corresponding non-IBC. Unfortunately, however, the external validation study failed to show the universality of the newly detected cutoff points on prognosis. In fact, the distribution of HR expression was significantly different between MD Anderson’s cohort and the validation cohort, showing 72.2 and 80.4% in mean ER and 40.4 and 53.8% in PR for MD Anderson’s cohort and the validation cohort, respectively. Accordingly, OS was generally better in the validation cohort than in the MD Anderson cohort (data not shown). The difference in survival was probably due to the fact that most of the patients with IBC at MD Anderson were referred from community clinics and this data set included more complexed or advanced cases with comorbidities. In addition, the difference in diagnostic criteria for IBC could affect the outcome. Further investigation is needed to establish the globally applicable cutoff point.

In the GE analysis, *MYC* was found to be upregulated in 3 of 4 pathways overlapping the ER pathway, and the gene had a significant impact on survival outcome in IBC. *MYC* is a regulator gene coding for transcriptional factors involved in cell cycle and cell growth. Generally, *MYC* amplification was observed in more aggressive subtypes such as HER2+ and triple-negative types [14], as well as in advanced clinical status [15], leading to poor survival outcome [16, 17]. For IBC, *MYC* has been investigated mainly in the triple-negative type [18, 19]; however, the present study found that *MYC* was also upregulated in HR+/HER2– IBC, leading to a significant association with poor survival outcome.

Generally, *MYC* expression was associated with cell cycle activity with increased cyclin B1 and Ki-67 expression [17] and can be a predictive marker for endocrine therapy resistance [20]. Indeed, we observed *MYC* upregulation in the ERK/MAPK and PDGF pathways, which have a significant role in endocrine therapy resistance [21, 22]. The activation of ERK/mitogen-activated protein kinase induces tamoxifen resistance by altering the level of estrogen-related receptor γ (ERRγ), which is an orphan member of the nuclear receptor superfamily. Furthermore, ERRγ-driven transcriptional activity is impaired by the mutation of ERK target sites, leading to the tamoxifen resistance [21]. For the PDGF pathway, a clinical study of 45 breast cancer patients treated with an aromatase inhibitor showed that the protein expression of PDGF receptor α and β in tumor was significantly increased at the point of relapse and the higher expression was correlated with shorter time to treatment failure [22]. Although the detailed mechanism for endocrine therapy resistance by *MYC* for HR+/HER2– IBC needs to be further investigated, the results in the present study suggest that *MYC* possibly contributed to poor prognosis due to either intrinsic characteristics or endocrine treatment resistance.

Notably, *MYC* upregulation contributed to survival outcome only in RFS and DDFS but not in OS for HR+/HER2– IBC. Previous studies had suggested that IBC has a unique metastatic process characterized by higher lymphatic invasion, tumor embolization, activated inflammatory pathways, and increased growth factors [23]. The *MYC* gene codes transcription factors and regulates every stage of the metastasis process, including cell proliferation, angiogenesis, and epithelial-to-mesenchymal transition [24]. However, it is unclear whether *MYC* has any specific effect on the metastatic process, especially for IBC. We reported that metastasis for IBC was associated with a risk allele at 8q24 where *MYC* located [25]. Moreover, we determined that the *MYC* activation in IBC was caused by the dysfunctional antagonization of *MYC* by the activation of *SMAD3*, which was located downstream of the TGF-beta signaling pathway [26]. Since *MYC* can be activated by upstream signaling pathways and codes many transcriptional factors, more comprehensive gene analysis will be needed to elucidate how *MYC* affects the metastasis process in HR+/HER2– IBC.

The chief limitation of the present study is that we excluded a certain number of patients during the selection process because they did not have a detailed pathological report; most of these patients had been evaluated outside of MD Anderson. Although we cannot estimate the result of excluding these patients, it is possible that HR distribution and the cutoff point may have been different if all cases had been included in the analysis. Moreover, the antibody used for IHC and the definition of HER2 positivity was not consistent over the study period, which possibly affected the overall results.

## Conclusions

The present study was the first to find that higher ER expression level was significantly associated with better survival, even in patients with HR+/HER2– IBC. Gene analysis showed that IBC had several activated pathways with *MYC* upregulation compared with corresponding non-IBC. The results indicated that *MYC* may be a key gene for understanding the biological behavior of HR+/HER2– IBC.

## Supplementary information

**Additional file 1: Figure S1.** CONSORT diagram. **Figure S2.** Scatter plot of ER% and PR% in IBC and non-IBC. **Figure S3.** Result of external validation for newly defined cutoff points by the cohort from Institut Paoli-Calmettes. **Figure S4.** Top canonical pathways including upregulated/downregulated genes associated with IBC by ingenuity pathway analysis. After analyzing the 84 genes associated with IBC with use of ingenuity pathway analysis, the top 15 canonical pathways, which included those genes, were revealed. The *z-*score determines whether an upstream transcription regulator has significantly more “activated” predictions than “inhibited” predictions (*z* > 0) or vice versa (*z* < 0). The ratio in the figure means the percentage of included genes in each pathway. All *P* values were unadjusted.

**Additional file 2: Table S1.** Patient background in gene expression analysis. **Table S2.** Probes and symbols for gene expression analysis. **Table S3.** Upregulated and downregulated genes in four pathways overlapping the estrogen receptor signaling pathway. **Table S4.** The result of the Cox proportional hazard model based on *MYC* expression in ER+/HER2– IBC. Hazard ratio was calculated according to the continuous value of *MYC* expression.

## Data Availability

All analyzed data are included in this published article and its supplementary file. The original data are available upon reasonable request to the corresponding author.

## Abbreviations

DDFS: Distant disease-free survival
ER: Estrogen receptor
GE: Gene expression; ERK, extracellular signal-regulated kinase
HER2: Human epidermal growth factor receptor 2
HR: Hormone receptor
HR +: Hormone receptor–positive.
HR–: Hormone receptor–negative
IBC: Inflammatory breast cancer;
IHC: Immunohistochemical
IL-7: Interleukin-7
MAPK: Mitogen-activated protein kinase
NAC: Neoadjuvant chemotherapy
PDGF: Platelet-derived growth factor
RFS: Recurrence-free survival
OS: Overall survival
PR: Progesterone receptor
RPA: Recursive partitioning analysis
