# Supplementary material for: Quantitative hormone receptor (HR) expression and gene expression analysis in HR+ inflammatory breast cancer (IBC) vs non-IBC
Source: BMC Cancer. 2020 May 18;20:430. doi: 10.1186/s12885-020-06940-z (PMC7236459; doi:10.1186/s12885-020-06940-z)

**SUPPLEMENTERY FIGURES**

**Supplementary Figure 1.** CONSORT diagram

**
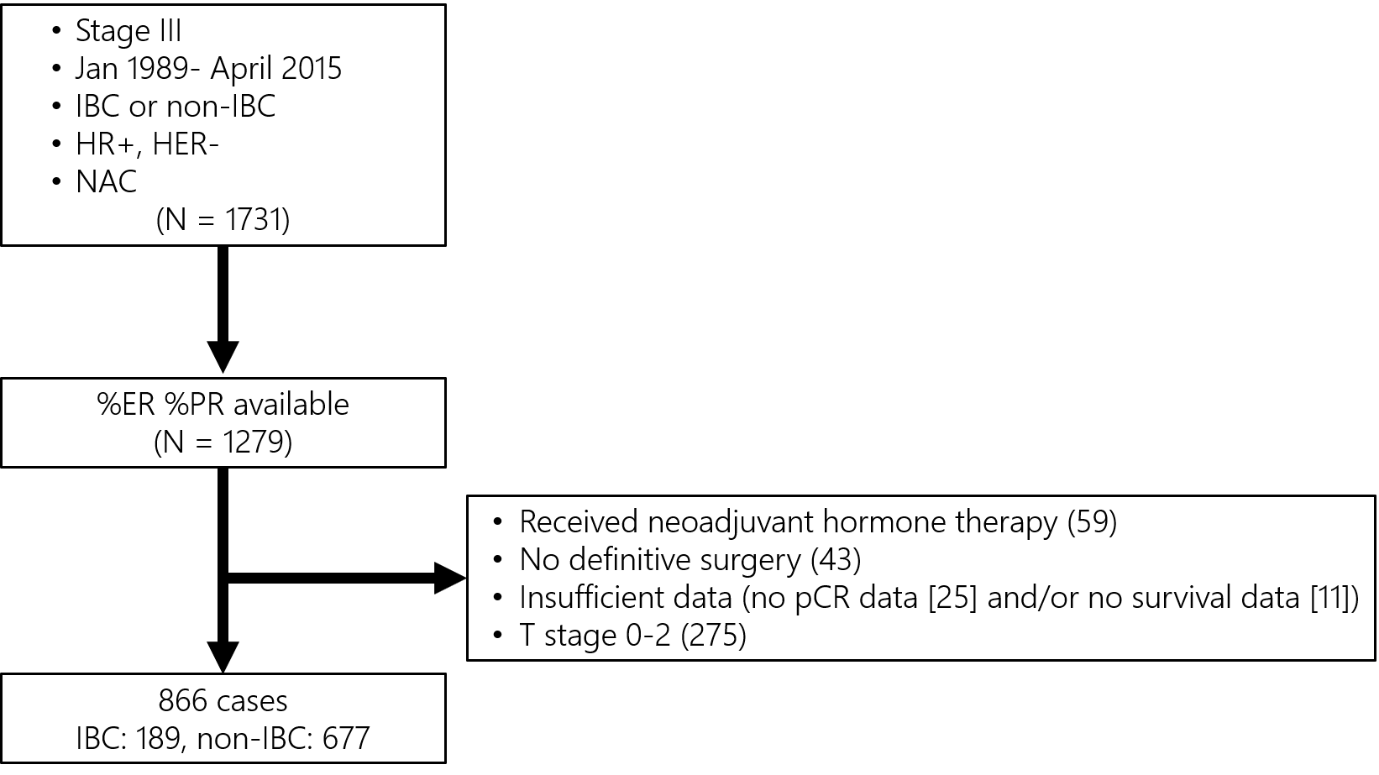
**

**Supplementary Figure 2.** Scatter plot of ER% and PR% in IBC and non-IBC

IBC, N = 189

non-IBC, N = 677

**Supplementary Figure** **3.** Result of external validation for newly defined cutoff points by the cohort from Institut Paoli-Calmettes.

1. Distant disease-free survival by ER and PR levels for non-IBC





1. Distant disease-free survival by ER and PR levels for IBC





1. Overall survival by ER and PR levels for non-IBC





1. Overall survival by ER and PR levels for IBC

**

**

**Supplementary Figure 4.** Top canonical pathways including upregulated/downregulated genes associated with IBC by ingenuity pathway analysis.


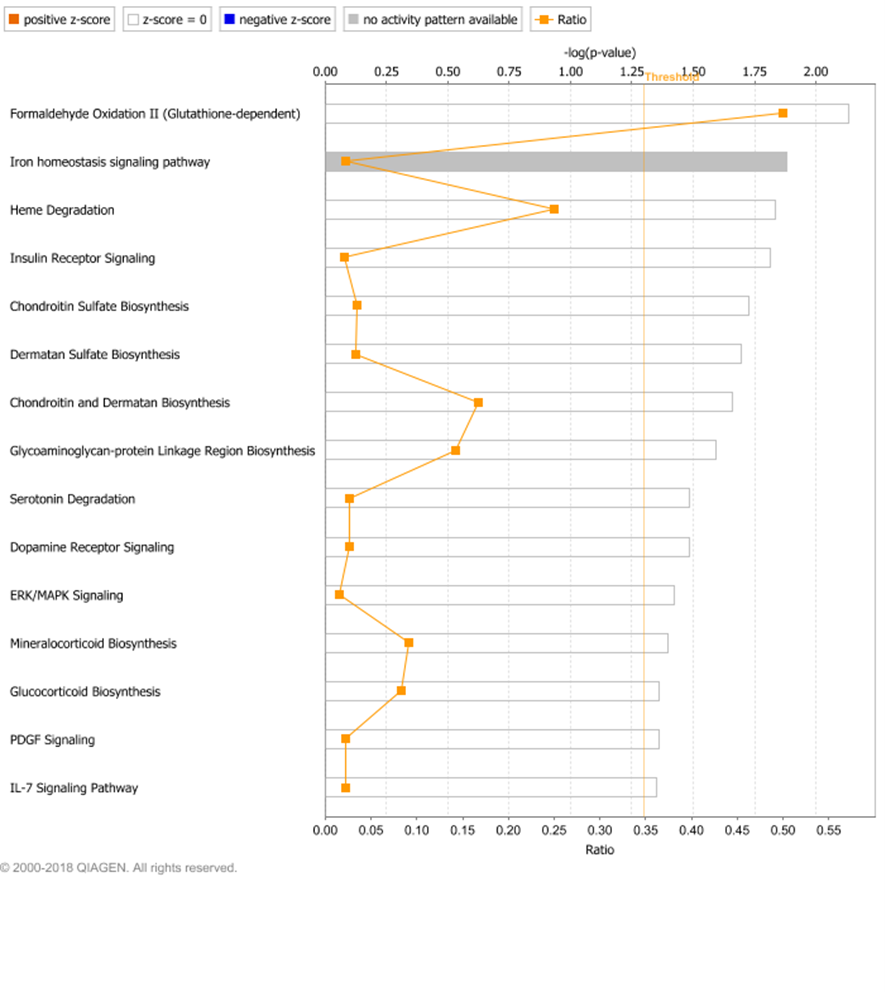

Supplement: Supplementary file 1 — Additional file 1: Figure S1. CONSORT diagram. Figure S2. Scatter plot of ER% and PR% in IBC and non-IBC. Figure S3. Result of external validation for newly defined cutoff points by the cohort from Institut Paoli-Calmettes. Figure S4. Top canonical pathways including upregulated/downregulated genes associated with IBC by ingenuity pathway analysis. After analyzing the 84 genes associated with IBC with use of ingenuity pathway analysis, the top 15 canonical pathways, which included those genes, were revealed. The z-score determines whether an upstream transcription regulator has significantly more “activated” predictions than “inhibited” predictions (z > 0) or vice versa (z < 0). The ratio in the figure means the percentage of included genes in each pathway. All P values were unadjusted. [file 12885_2020_6940_MOESM1_ESM.docx]
