# Supplementary material for: Quantitative hormone receptor (HR) expression and gene expression analysis in HR+ inflammatory breast cancer (IBC) vs non-IBC
Source: BMC Cancer. 2020 May 18;20:430. doi: 10.1186/s12885-020-06940-z (PMC7236459; doi:10.1186/s12885-020-06940-z)
Supplement: Supplementary file 2 — Additional file 2: Table S1. Patient background in gene expression analysis. Table S2. Probes and symbols for gene expression analysis. Table S3. Upregulated and downregulated genes in four pathways overlapping the estrogen receptor signaling pathway. Table S4. The result of the Cox proportional hazard model based on MYC expression in ER+/HER2– IBC. Hazard ratio was calculated according to the continuous value of MYC expression. [file 12885_2020_6940_MOESM2_ESM.docx]

**SUPPLEMENTARY TABLES**

**Supplementary Table 1.** Patient background in gene expression analysis

| Characteristic | IBC  (n = 39) | % |  | non-IBC  (n = 40) | % |  | *P* |
| --- | --- | --- | --- | --- | --- | --- | --- |
| Age (median, range) (years) | 52 (24-82) |  |  | 57 (30-80) |  |  | 0.111 |
| Histology |  |  |  |  |  |  |  |
| Ductal | 34 | (87) |  | 35 | (87) |  | 0.651 |
| Lobular | 4 | (10) |  | 3 | (7) |  |  |
| Mixed | 1 | (3) |  | 1 | (3) |  |  |
| Other | 0 | (0) |  | 1 | (3) |  |  |
| Nuclear Grade |  |  |  |  |  |  |  |
| 1 | 0 | (0) |  | 3 | (7) |  | 0.169 |
| 2 | 18 | (46) |  | 17 | (43) |  |  |
| 3 | 19 | (49) |  | 20 | (50) |  |  |
| Unknown | 2 | (5) |  | 0 | (0) |  |  |

Abbreviation: IBC, inflammatory breast cancer

**Supplementary Table 2.** Probes and symbols for gene expression analysis

| Probe set | Symbol | Probe set | Symbol | Probe set | Symbol |
| --- | --- | --- | --- | --- | --- |
| 214955_at | TMPRSS6 | 203994_s_at | C21orf2 | 213412_at | TJP3 |
| 219310_at | TMEM90B | 214622_at | CYP21A2 | 220003_at | LRRC36 |
| 204144_s_at | PIGQ | 220587_s_at | MLST8 | 203510_at | MET |
| 218537_at | HCFC1R1 | 201703_s_at | PPP1R10 | 219382_at | SERTAD3 |
| 219051_x_at | METRN | 206076_at | LRRC23 | 218812_s_at | ORAI2 |
| 212823_s_at | PLEKHG3 | 221986_s_at | KLHL24 | 45714_at | HCFC1R1 |
| 218931_at | RAB17 | 213771_at | IRF2BP1 | 216663_s_at | ZMYND10 |
| 219172_at | UBTD1 | 210021_s_at | CCNO | 205524_s_at | HAPLN1 |
| 208847_s_at | ADH5 | 209763_at | CHRDL1 | 205712_at | PTPRD |
| 219047_s_at | ZNF668 | 211889_x_at | CEACAM1 | 213651_at | INPP5J |
| 222172_at | NPAS3 | 220757_s_at | UBXN6 | 221957_at | PDK3 |
| 205252_at | ZNF174 | 221880_s_at | FAM174B | 205044_at | GABRP |
| 219483_s_at | PORCN | 209821_at | IL33 | 212383_at | ATP6V0A1 |
| 201884_at | CEACAM5 | 205896_at | SLC22A4 | 38043_at | FAM3A |
| 217066_s_at | DMPK | 217988_at | CCNB1IP1 | 220493_at | DMRT1 |
| 220947_s_at | TBC1D10B | 215047_at | TRIM58 | 217209_at | CEACAM3 |
| 218121_at | HMOX2 | 212066_s_at | USP34 | 206724_at | CBX4 |
| 216234_s_at | PRKACA | 202801_at | PRKACA | 219245_s_at | OGFOD2 |
| 222220_s_at | TSNAXIP1 | 202175_at | CHPF | 221603_at | PEX16 |
| 202219_at | SLC6A8 | 213823_at | HOXA11 | 41577_at | PPP1R16B |
| 210608_s_at | FUT2 | 215544_s_at | UBOX5 | 201446_s_at | TIA1 |
| 207031_at | NKX3-2 | 35179_at | B3GAT3 | 204986_s_at | TAOK2 |
| 211883_x_at | CEACAM1 | 50965_at | RAB26 | 215131_at | IQCK |
| 45526_g_at | NAT15 | 219223_at | C9orf7 | 213906_at | MYBL1 |
| 215843_s_at | TLL2 | 203838_s_at | TNK2 | 206685_at | HCG4 |
| 213781_at | LRRC68 | 214141_x_at | SRSF7 | 206638_at | HTR2B |
| 202030_at | BCKDK | 215130_s_at | IQCK | 202431_s_at | *MYC* |
| 208458_at | SCNN1D | 221564_at | PRMT2 | 222185_at | PKNOX2 |

**Supplementary Table 3.** Upregulated and downregulated genes in four pathways overlapping the estrogen receptor signaling pathway

| Pathway | Upregulated genes |  | Downregulated genes |
| --- | --- | --- | --- |
| ERK/MAPK signaling pathway | *MYC* |  | *PPP1R10* |
|  | *PRKACA* |  |  |
| PDGF signaling pathway | *MYC* |  |  |
|  | *INPP5J* |  |  |
| Insulin receptor signaling pathway | *PPP1R10* |  | *INPP5J* |
|  |  |  | *PRKACA* |
| IL-17 signaling pathway | *MYC* |  |  |
|  | *MET* |  |  |
| Abbreviations: ERK, extracellular signal-regulated kinases; MAPK, mitogen-activated protein kinases; PDGF, platelet-derived growth factors; IL-17, interleukin-17 | | | |

**Supplementary Table 4.** The result of the Cox proportional hazard model based on *MYC* expression in ER+/HER2– IBC. Hazard ratio was calculated according to the continuous value of *MYC* expression.

| Outcome |  | IBC | | | | |  |
| --- | --- | --- | --- | --- | --- | --- | --- |
|  |  | HR | 95% CI | | | *P* |  |
| Relapse-free survival |  | 1.93 | 1.09 | to | 3.43 | 0.003 |  |
| Distant disease-free survival |  | 2.00 | 1.10 | to | 3.64 | 0.028 |  |
| Overall survival |  | 1.45 | 0.65 | to | 3.24 | 0.38 |  |
| HR, hazard ratio; 95% CI, 95% confidence interval; IBC, inflammatory breast cancer | | | | | | | |
